# Population Structure and Ecological Niches of Benthic Macroinvertebrates in the Upper Yarlung Zangbo River

**DOI:** 10.3390/biology14111604

**Published:** 2025-11-16

**Authors:** Zepeng Zhang, Hongyu Jin, Shenhui Li, Haipeng Wang, Shitong Xing, Wanqiao Lu, Lei Li

**Affiliations:** 1 Heilongjiang River Fisheries Research Institute of Chinese Academy of Fishery Sciences, Heilongjiang River Basin Fishery Resources and Environment Scientific Observation and Experiment Station of the Ministry of Agriculture and Rural Affairs, Harbin 150070, China; zhangzepeng@hrfri.ac.cn (Z.Z.);; 2 College of Fisheries and Life Sciences, Shanghai Ocean University, Shanghai 201306, China

**Keywords:** benthic macroinvertebrate, Yarlung Zangbo River, beta-diversity, ecological niche width, ecological niche overlap, diversity

## Abstract

The community structure and ecological niche of benthic macroinvertebrates in the upper Yarlung Zangbo River were analyzed in April and September 2023. The benthic macroinvertebrate community largely comprises aquatic insects, with Diptera accounting for approximately half. Commonly observed were *Chironomus anthracinus*, *Tadamus* sp.1, *Piscicola geometra*, species of the family Corixidae spp. and the genera *Monodiamesa* sp., *Apatania* sp., and *Valvata* sp. in April, and *Orthocladius* sp.1, *Gammarus* sp., *Isoperla* sp., *Nais* sp., *Baetis* sp., *Monodiamesa* sp., *Tanytarsus* sp., *Ilisia* sp., *Nebrioporus* sp. and species of the family Corixidae spp. in September. The α-diversity analysis showed significant seasonal differences (*p* < 0.05) in Shannon–Wiener diversity variable and Margalef richness variable. The Pielou evenness variable did not show seasonal effects (*p* > 0.05). The β-diversity April/September differences can be attributed to species turnover rather than to nestedness, indicating that benthic macroinvertebrate diversity protection strategies are critical to all areas of the river. In April, *Chironomus anthracinus* exhibited the broadest ecological niche, and, in September, the widest niche was observed in *Gammarus* sp. The largest observed ecological niche overlap values were between *Chironomus anthracinus* and *Valvata* sp. in April and *Gammarus* sp. and *Ilisia* sp. in September, indicating interspecific competition. The study clarifies the diversity status of benthic macroinvertebrates in the upper Yarlung Zangbo River and provides data for related research to facilitate formulation of biodiversity conservation policies.

## Simple Summary

As natural prey for benthic or omnivorous fish, macroinvertebrates play a vital role in facilitating energy transfer and material cycling within aquatic ecosystems through their connections within the food web, thereby significantly contributing to the maintenance of normal ecosystem functioning. Through our field surveys and data analysis of macroinvertebrate communities in the upper reaches of the Yarlung Zangbo River (at elevations exceeding 4500 m), conservation strategies for macroinvertebrates diversity prove crucial for all regions within the upper reaches. It is recommended to enhance aquatic ecosystem conservation efforts, reduce pollution, and provide food sources for rare cold-water fish species.

## 1. Introduction

The Yarlung Zangbo River is the longest plateau river in China and one of the world’s highest rivers, at elevation exceeding 4500 m. It drains a broad area of China, India, and Bangladesh and is ecologically, economically, and politically important [1,2]. The topography of the Yarlung Zangbo River basin is complex, with the terrain of its upper reaches dominated by plateaus and mountains. Its middle reaches cross the Himalayas, carving deep gorges and valleys. At lower elevations it enters the South Asian plains, where the terrain gradually flattens and the flow slows [3]. The Yarlung Zangbo River and its watershed nurture a range of endemic and rare wildlife and is a hotspot of global biodiversity research [4,5,6]. The extreme climate conditions and unique geographic features of the upper Yarlung Zangbo River make it an ideal region for study of biological adaptation and ecology.

Flow velocity, dissolved oxygen content, and temperature fluctuations are the fundamental factors affecting biological communities of high elevation aquatic ecosystems [7,8]. As a critical component of river ecosystems, benthic macroinvertebrates play a key role in material cycling and energy flow [9,10]. They represent an important link in the aquatic food web and are key indicators of water quality [11]. Most available studies of benthic macroinvertebrates have been conducted at low elevation or in temperate regions, and little is known about their ecological function in alpine environments. The upper reaches of the Yarlung Zangbo River, at elevation above 4500 m asl, have long been threatened by climate change and natural disasters, and research on aquatic organisms in this region is particularly lacking [12,13,14]. Information of the community structure and spatial and temporal diversity of benthic macroinvertebrates in the upper reaches of the Yarlung Zangbo River can provide a basis for conservation of its ecosystem and biodiversity.

Alpha diversity (*α*-diversity) refers to the number of species and their relative abundance in a given area. Parameters of alpha diversity include species richness (the number of species in the area) and species evenness (the distribution of individuals of each species within the community) [15]. Beta diversity (*β*-diversity) represents the degree of difference in species community composition among local sites within the larger area, potentially related to environmental factors or spatial variation. It is measured as species turnover and as nestedness [16]. Species turnover (*β_jtu_*) refers to the degree of species replacement among locations, while nestedness (*β_jnc_*) indicates differences in richness of communities, i.e., whether species assemblages contained in one community are fully present in other communities [17,18,19]. Ecological niche theory is a basic hypothesis in ecology and plays a critical role in the study of community structure and function, interspecific relationships, biodiversity, community dynamic succession, and population evolution. The ecological niche analysis is a valuable tool for the study of how benthic macroinvertebrate survive, reproduce, and interact with their environment. Species ecological niche is influenced by the biotic and abiotic environment within the community and impacts interspecific competition [20]. Ecological niche theory can help explain why certain habitats support high alpha diversity [15]. Niche differentiation is a critical driver of species beta diversity, especially along environmental gradients [19,21]. Overlapping ecological niches may lead to competitive exclusion, affecting species distribution patterns [22].

Steep drops in elevation lead to rapid flow in the upper reaches of the Yarlung Zangbo River, creating a turbulent river environment, especially through the canyon sections. The upper reaches are located in a high mountain valley area with rugged topography and extensive erosion, resulting in low habitat stability. Here, “habitat stability” refers to the ability of a habitat to maintain its physical structure (e.g., riverbed, banks, flow regime) and ecological functions over time and space. Low stability implies frequent hydrological disturbances, intense erosion and sedimentation processes, which make species colonization difficult and lead to frequent community turnover. This study aims to investigate the alpha and beta diversity characteristics, population structure composition and dynamics of benthic macroinvertebrate communities in the upper reaches of the Yarlung Zangbo River (elevations exceeding 4500 m) through systematic field surveys and data analysis for the first time, and to explore the ecological niche characteristics of common taxa. By revealing the distribution patterns and ecological adaptation mechanisms of these biological communities in the unique high-altitude environment, this study can provide a basis for conserving benthic species diversity in the Yarlung Zangbo River Basin and similar high-elevation rivers.

## 2. Materials and Methods

### 2.1. Study Area

The upper reaches of the Yarlung Zangbo River flow through the northern foothills of the Himalayas, primarily in the southern Tibet Autonomous Region of China, from Jemayangzong Qu, its primary source in Zhongba County, to the Lizi section, extending ~268 km, 13% of its total length. The area is at elevation above 4600 m asl and has an alpine climate with cold weather and generally low precipitation but is provided abundant precipitation by the Indian monsoon in summer [23]. This section of the river traverses from west to east, through broad valleys, forming a unique plateau geomorphology and ecosystem [24].

### 2.2. Sampling Sites and Sample Collection

**Figure 1:**
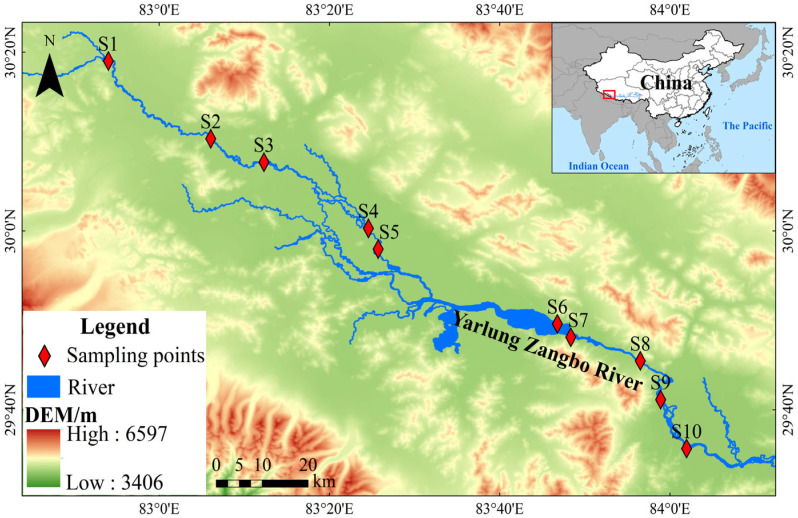
Sampling sites for benthic macroinvertebrates in the upper Yarlung Zangbo River.

Due to limitations in transportation and fieldwork conditions in high-elevation regions, 10 sampling sites were selected in Zhongba County, at mean elevation of >4500 m (Figure 1). Sampling was conducted once in late April (spring at this latitude) and late September (late summer/early autumn at this latitude) of 2023, aiming to balance spatial coverage and seasonal dynamics.

A long-handled (2 m) square kick net (30 × 30 cm) was used for collection of benthic macroinvertebrates. The collection area was limited to within 1 m of the riverbank. The substrate in front of the net frame was disturbed by kicking and the net was moved against the current for three meters (total area of 1 m^2^), so that aquatic insects, crustaceans, and other benthic macroinvertebrates flowed into the net. The samples were filtered through a 0.425 mm mesh sieve [25]. Specimens were placed individually in 100 mL plastic bottles according to sampling site and fixed in 10% formalin solution. In the laboratory, the specimens were identified by a stereomicroscope (Nikon SMZ745T, Nikon Ltd., Tokyo, Japan) with an objective zoom ratio ≥ 5:1 and an eyepiece of 10× or 15×. Specimens were identified to the lowest possible taxonomic level, typically genus or species, based on appropriate identification guides [26,27,28]. Specimens were counted and weighed (fresh weight) to calculate abundance and biomass within each sampling area.

### 2.3. Data Analysis

#### 2.3.1. α-Diversity

The Shannon–Wiener diversity (*H*′), Margalef richness (*D*), and Pielou evenness (*J*) variables were calculated to analyze the α-diversity characteristics of the benthic macroinvertebrate population as

*H*′ = −∑(*P_i_*)(log_2_*P_i_*)

*D* = (*S* − 1)/ln*N*

*J* = *H*′/log_2_*S*

where *S* is the number of taxa, *N* is the number of individuals of each taxon in a sample, and *P_i_* is the relative abundance of taxa [29,30,31].

#### 2.3.2. *β*-Diversity

*β*-diversity represents the level of difference of composition among communities. The *β*-diversity (*β_jac_*) was calculated by Jaccard’s dissimilarity index using the “adespatial” package in R 4.2.1 software and categorized as *β_jtu_* (turnover) and *β_jnc_* (nestedness). Results were plotted in ternary diagrams of *β*-diversity using the “ggplot” package in R 4.2.1 software [19,32] with each point representing a pair of sampling sites of which the position is determined by the mean of values of the similarity (1-dissimilarity index), species turnover, and nesting matrices, with the sum of each ternary factor equal to 1. The relevant formulae are

*β_jac_* = 1 − *a*/(*a* + *b* + *c*)

*β_jac_* = *β_jtu_* + *β_jnc_*

where *a* is the number of taxa shared among sampling sites, and *b* and *c* are the number of taxa endemic to each sampling site.

#### 2.3.3. Ecological Niche

We classified species with >10% frequency of occurrence at all sampling sites and ≥1% relative abundance in at least one site as common taxa. The ecological niche width (*B_i_*) and ecological niche overlap index (*O_ik_*) were calculated to analyze the ecological niche status of common taxa.

*B_i_* was measured based on the Shannon–Wiener diversity index [29]:

$$B_{i}=-\sum_{j=1}^{R}P_{ij}\ln P_{ij}$$

*O_ik_* was measured using the Pianka index [33]:

$$Q_{ik}=\sum_{j=1}^{R}(P_{ij}P_{kj})/(\sqrt{\sum_{j=1}^{R}P_{ij}^{2}}\sqrt{\sum_{j=1}^{R}P_{kj}^{2}})$$

where *R* is the number of sampling sites, and *P_ij_* and *P_kj_* are the proportion of individuals of taxa *i* and *k* in sampling site *j*. *B_i_* is in the range of 0–ln*R*, with higher values representing wider ecological niche. The *O_ik_* ranges from 0–1, with *O_ik_* > 0.3 regarded as meaningful overlap and the *O_ik_* > 0.6 as significant overlap.

### 2.4. Statistical Analysis and Mapping

One-way analysis of variance (ANOVA) using SPSS 26.0 software was conducted to compare differences in *α*-diversity indices of the benthic macroinvertebrate communities in the upper Yarlung Zangbo River between the two sampling periods. The normality of data in each group was checked using the Shapiro–Wilk test, and homogeneity of variance was assessed using Levene’s test before analysis. Only when the data meet the assumptions of normal distribution and homogeneity of variance are the results of ANOVA considered reliable). To explore the inter-relationships in abundance among common taxa, the “plotnetwork” function from the “spaa” package in R 4.2.1 software was used to construct Pearson correlation networks of common taxa between the two sampling periods. Spatial distribution mapping of the sampling sites was conducted in Arcgis 10.8 software. Basic data statistical analysis and graphing were performed using Origin 2019 software.

## 3. Results

### 3.1. Community Characteristics

**Figure 2:**
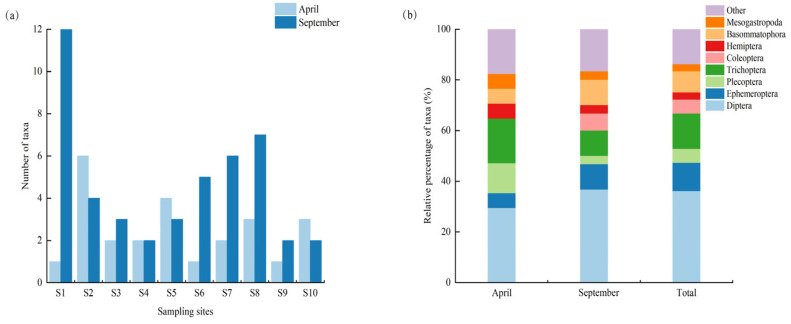
Number of taxa at each sampling site between the two sampling periods (**a**) and individual composition of benthic macroinvertebrates in each order (**b**). Note: *n* = 10 per season (1 sample per site).

A total of 209 benthic macroinvertebrate specimens (96 in April and 113 in September) representing 36 taxa (family, genus or species) of were collected during the two surveys (17 in April and 30 in September). The collected benthic macroinvertebrates belonged to 3 phyla, 5 classes, 12 orders, and 21 families (16.75% of individuals were identified to the species level, 71.29% to the genus level, and 11.96% to the family level). Aquatic insects were predominant among these taxa, comprising 27 taxa (which accounted for 75% of the total number of taxa), followed by 4 taxa of Mollusca (11.11%), 4 Annelida (11.11%), and 1 Crustacea (2.78%). Among aquatic insects, 13 taxa were Diptera, of which 11 taxa were larvae of the Chironomidae, 5 taxa were Trichoptera, 4 Ephemeroptera, 2 Coleoptera, 2 Plecoptera, and 1 Hemiptera. Aquatic insect taxa in general and Diptera in particular accounted for 70.59% and 41.67% of the total number of specimens, respectively, in April and 70% and 52.38% in September (Figure 2).

Taxa with a frequency of occurrence >10% at all sampling sites in April included *Chironomus anthracinus* (frequency of occurrence is 20%), *Tadamus* sp.1 (20%), and those belonging to the family Corixidae spp. (30%) and genera *Monodiamesa* sp. (30%), *Apatania* sp. (20%), and *Valvata* sp. (20%). In September, the taxa with the highest frequency of occurrence were *Piscicola geometra* (20%), *Orthocladius* sp.1 (20%), and species of the family Corixidae spp. (20%) and genera *Gammarus* sp. (60%), *Isoperla* sp. (30%), *Nais* sp. (20%), *Baetis* sp. (20%), *Monodiamesa* sp. (20%), *Tanytarsus* sp. (20%), *Orthocladius* sp.1 (20%), *Ilisia* sp. (20%), and *Nebrioporus* sp. (20%).

**Figure 3:**
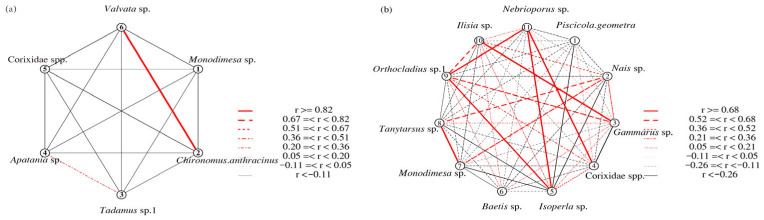
Pearson correlation network showing co-occurrence of common macroinvertebrate taxa in the upper Yarlung Zangbo River in April (**a**) and September (**b**), 2023. Note: A total of 57 specimens in April and 66 in September, *n* = 10 per correlation test.

The Pearson correlation network plot (Figure 3) shows that *Monodiamesa* sp. and *Valvata* sp. (r = 0.98) exhibited an extremely high correlation in abundance in April. In September, *Gammarus* sp. and *Ilisia* sp. (r = 0.83); Corixidae spp. and *Nebrioporus* sp. (r = 0.76); *Isoperla* sp., *Orthocladius* sp.1, and *Nebrioporus* sp. (r = 0.72); *Monodiamesa* sp. and *Tanytarsus* sp. (r = 0.80) show high correlation.

### 3.2. Abundance and Biomass

**Figure 4:**
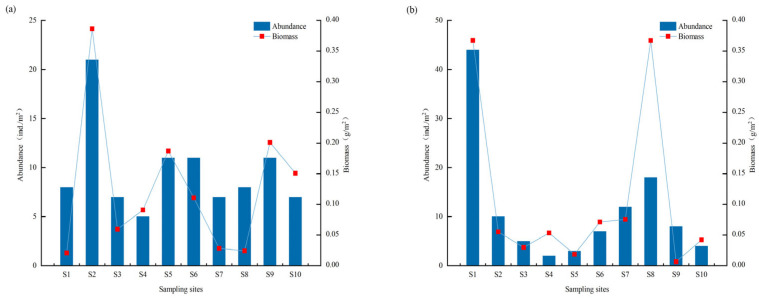
Abundance and biomass of benthic macroinvertebrates per sampling site in the upper Yarlung Zangbo River in April (**a**) and September (**b**), 2023. Note: *n* = 10 per season (1 sample per site).

The mean abundance of benthic macroinvertebrates in the upper Yarlung Zangbo River was 9.6 ind/m^2^ in April and 11.3 ind/m^2^ in September. The mean biomass of the benthic macroinvertebrates in the upper Yarlung Zangbo River was 0.13 g/m^2^ in April and 0.11 g/m^2^ in September. The upstream sites S1 and S2 showed highest abundance and biomass of benthic macroinvertebrates in September and April, respectively. Total abundance was higher in September than in April and total biomass was slightly lower based on original data (Figure 4).

### 3.3. Diversity Characteristics

#### 3.3.1. The *α*-Diversity of Benthic Macroinvertebrate Communities

**Figure 5:**
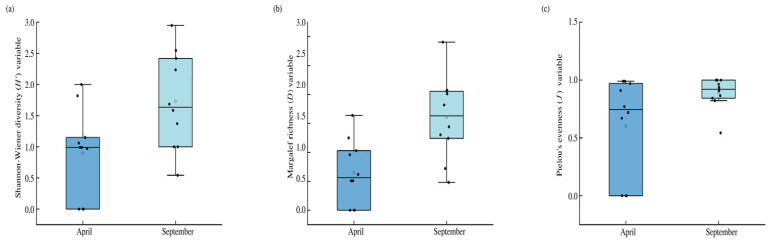
Seasonal *α*-diversity ((**a**): Shannon–Wiener diversity variable; (**b**): Margalef richness variable; (**c**): Pielou evenness variable) of the benthic macroinvertebrate community in the upper Yarlung Zangbo River. Note: Each box represents the interquartile range (IQR), with the median shown as a horizontal line inside the box. The whiskers extend to the minimum and maximum values within 1.5 × IQR from the quartiles. Black dots represent individual sample values, and the open square indicates the mean value for each group. Data are based on *n* = 10 samples per season.

A box plot of *α*-diversity of benthic macroinvertebrate communities in the upper Yarlung Zangbo River in April and September is shown in Figure 5. The Shannon–Wiener diversity (*H*′) variable ranged from 0 to 2.0 (mean 0.90) and 0.54 to 2.95 (mean 1.73) in April and September, respectively. The Margalef richness (*D*) variable was 0–1.64 (April) and 0.48–2.91 (September) with mean values 0.65 and 1.61, respectively. Pielou evenness (*J*) variable was 0–0.99 (April) and 0.54–1.0 (September) with mean values of 0.60 and 0.89, respectively (Table 1). One-way ANOVA results for the *α*-diversity of benthic macroinvertebrates in the basin showed significant seasonal differences in *H*′ and *D* (*p* = 0.023 and *p* = 0.004, respectively). Pielou evenness variable (*p* = 0.061) did not show a seasonal difference.

#### 3.3.2. The *β*-Diversity and Turnover/Nesting Patterns

**Figure 6:**
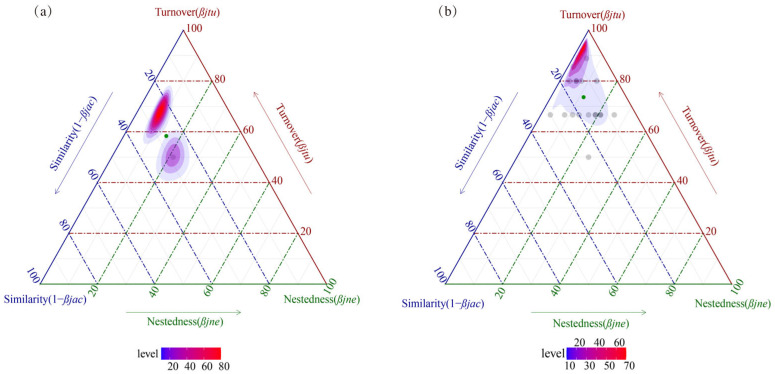
*β*-diversity triplex map of benthic macroinvertebrate communities in the upper Yarlung Zangbo River in April (**a**) and September (**b**), 2023. Note: The colors dots represent the *β*-diversity data for each pair of sampling sites.

The total *β*-diversity of the upper Yarlung Zangbo River quantified by Jaccard’s dissimilarity index was 0.73 in April and 0.85 in September and was further categorized as replacement (turnover) and differences in richness (nestedness) (Figure 6). In both sampling periods, the sites showed high replacement values, with replacement contributing 79.67% in April and 86.54% in September to community *β*-diversity.

### 3.4. Ecological Niche of Common Taxa

#### 3.4.1. Ecological Niche Width of Common Taxa

The ecological niche width of common benthic macroinvertebrate taxa in the upper Yarlung Zangbo River ranged from 0.32 to 0.71 in April and 0.26 to 1.87 in September (Table 2). In April, *Chironomus anthracinus* occupied the broadest ecological niche, followed by *Valvata* sp., showing capability of utilizing a wide range of resources. In September, *Gammarus* sp. exhibited the widest niche, followed by *Piscicola geometra*. The lower niche width values of the other taxa indicate their specialization in utilizing resources, with obvious seasonal and environmental selectivity.

#### 3.4.2. Ecological Niche Overlap of Common Taxa

The ecological niche index overlap values of common benthic macroinvertebrates ranged from 0 to 0.98 in April, with largest overlap in *Chironomus anthracinus* larvae and *Valvata* sp. (Table 3). In September, overlap values ranged from 0 to 0.85: 0.67 for *Nais* and *Tanytarsus* sp.; 0.62 for *Nais* sp. and *Monodiamesa* sp.; 0.67 for *Gammarus* sp. and *Orthocladius* sp.1; 0.85 for *Gammarus* sp. and *Ilisia* sp.; 0.80 for Corixidae spp. and *Nebrioporus* sp.; 0.77 for *Isoperla* sp. and *Orthocladius* sp.1; 0.77 for *Isoperla* sp. and *Nebrioporus* sp.; 0.84 for *Monodiamesa* sp. and *Tanytarsus* sp.; 0.63 for *Orthocladius* sp.1 and *Ilisia* sp.; and 0.80 for *Orthocladius* sp.1 and *Nebrioporus* sp. (Table 4). Ecological niche index overlap values of 0 indicate that complete separation of ecological niche.

## 4. Discussion

Aquatic insects comprise the dominant taxa of benthic macroinvertebrates in the upper Yarlung Zangbo River. This is similar to the situation in the lower reaches of the Yarlung Zangbo River [14]. According to Chi et al. [34], the benthic macroinvertebrate community of the Jinsha River Basin is also dominated by aquatic insects, which is related to their short life cycle and ability to compensate for the extreme environment of the plateau by diapausing eggs. Aquatic insects can rapidly colonize newly available habitat patches and become the dominant population in unpolluted water [35]. In contrast, the dominant benthic macroinvertebrate taxa in some heavily polluted rivers are reported to be Mollusca and annelids [36]. The larvae of Ephemeroptera, Plecoptera, and Trichoptera (EPT) insects inhabit clear-water streams and are sensitive to pollution, making them suitable biomarkers for monitoring water quality [37]. The numerous EPT insects present in the upper Yarlung Zangbo River reflects its unpolluted state. This has been verified in high-elevation lakes and rivers [38]. Diptera accounted for a high proportion of aquatic insects observed. This can be attributed to *Chironomus* larvae preference for slow-flowing areas along riverbanks with abundant sediment deposits [17] characteristic of to our sampling sites. We collected four taxa of Mollusca (i.e., *Gyraulus convexiusculus*, *Radix swinhoei*, *Radix ovata*, and *Valvata* sp.), which typically require warmer waters and more abundant food sources than found in the upper Yarlung Zangbo River [39]. *Gammarus* sp. was more common in September than April, when water levels are high than in April, which may be a consequence of drift of tributary populations into the main stream and subsequent dispersal [17]. This pattern may be driven by two ecological mechanisms: (1) frequent precipitation in September increases tributary flow, enhancing the scouring effect on benthic macroinvertebrates and promoting passive drift of *Gammarus* sp. into the main stream; (2) *Gammarus* sp. larvae reach maturity in September and may migrate to the main stream through active dispersal to expand their distribution range.

The biomass and abundance of benthic macroinvertebrates in the upper Yarlung Zangbo River is low. We found the total mean abundance lower than reported in the Xiongcun reach (2007) but slightly higher total mean biomass [40]. This may be attributed to the contribution of larger-bodied individuals (such as Mollusca), rather than an increase in the number of individuals. Our study area was located in Zhongba County, near the headwaters, where water velocity and low nutrient input may lead to a scarcity of fauna, especially in spring.

There were significant seasonal differences in the Shannon–Wiener diversity and Margalef richness variables (*p* < 0.05) but not in Pielou evenness variable (*p* > 0.05). Although not measured in the current study, water quality is an important factor that drives macroinvertebrate community patterns [41,42,43]. Zeng et al. [41] found correlation of dissolved oxygen with Pielou evenness variable in their study of the Jiulong River estuary. The lack of pronounced seasonal variation in Pielou evenness in this study may suggest that dissolved oxygen levels were relatively stable between the two sampling periods, or that fluctuations did not reach the threshold necessary to affect community evenness. Zhao et al. [42] reported that the Margalef richness variable was significantly positively correlated with NH_3_-N content in rivers of the southern mountainous area of Jinan City. This suggests that nutrient conditions may influence species richness. Considering that alpine rivers are typically less affected by anthropogenic pollution, we believe that the seasonal variation in richness observed in this study is more likely regulated by natural environmental factors. Elevation may be an important environmental factor affecting the *α*-diversity of benthic macroinvertebrates. Environmental conditions such as dissolved oxygen, water temperature, and solar intensity differ with elevation [43]. In addition, water temperature and hydrological regimes (such as flow variations caused by snowmelt) exhibit pronounced seasonal patterns in rivers of the Tibetan Plateau [44]. We speculate that the observed seasonal differences in Shannon–Wiener diversity and Margalef richness in this study may be primarily driven by seasonal changes in water temperature and alterations in hydrodynamic conditions. Our findings provide a baseline reference for future studies on environment-biota coupling analyses.

*β*-diversity refers to the degree of difference in species community composition among habitats or sampling sites and reflects the spatial succession and distribution patterns of species [45]. We found *β*-diversity levels of the benthic macroinvertebrate communities in the upper Yarlung Zangbo River to be high, indicating significant differences in local fauna community structure. With respect to preserving biodiversity, when total *β*-diversity can be attributed disproportionally to nestedness, sites with the greatest number of species may have higher conservation priority. If species turnover is the dominant factor, i.e., all sites contribute equally to the *β*-diversity, all species at all sites require protection [18,19]. We found the turnover process of the benthic macroinvertebrate communities to have a decisive impact on *β*-diversity. This may be related to the filtering effect of the environment on the community structure of sampling sites. Environmental filtering impacts species turnover [17] and plays an essential role in the structure of benthic macroinvertebrate communities and in maintaining community dynamics. By integrating stream data from Europe and North America, it has been shown that natural environmental gradients (such as elevation, stream width, and flow velocity) exert strong filtering effects on the functional traits of fish and benthic macroinvertebrates, leading to significant species turnover along these gradients [46]. Future research should explore the interaction between environmental filtration (such as water temperature, flow velocity, organic matter input, and other factors) and diffusion limitations as well as seasonal and spatial variation.

Ecological niche width refers to the breadth and diversity of resource utilization by a species in a given environment. It is an important indicator of species adaptability and resource utilization efficiency. A broad ecological niche represents high adaptability and the potential to utilize a wide variety of resources. This generally means stronger competitive ability within the community [47]. The macroinvertebrate community structure of the upper Yarlung Zangbo River was shown to be simple, with aquatic insects being the major component. These species typically occupy a narrow ecological niche with specific requirements for environmental conditions such as water quality, flow velocity, and temperature and are unable to disperse widely in varied environments [13]. Li et al. [13] and Zhang et al. [14] reported low diversity of benthic macroinvertebrates in the middle and lower reaches of the Yarlung Zangbo River and a simple community structure primarily influenced by elevation, flow velocity, river width, and substrate type.

Ecological niche overlap can describe the competitive relationship among species. We found low overlap among common taxa, with only *Chironomus anthracinus* and *Valvata* sp. showing significant overlap in April. Both species feed primarily on organic debris and similar substances, indicating high niche similarity and the potential for competitive pressure. However, under conditions of relatively abundant resources, species may coexist without exhibiting intense competitive interactions.

Changes in weather such as lower temperatures and reduced precipitation in September may affect available food and habitat in the upper Yarlung Zangbo River. Some common taxa showed no ecological niche overlap in September, indicating that, although these species inhabit the same geographic area, ecological niche differentiation, such as vertical structure, horizontal structure, and differences in feeding habits, reduces competition [48].

## 5. Conclusions

Although the number of samples is limited, this trend still reflects the characteristics of seasonal changes in community structure. The benthic macroinvertebrate communities in the upper Yarlung Zangbo River are dominated by aquatic insects, with richness showing a pattern of September > April. Species turnover is the dominant process influencing the diversity of the benthic macroinvertebrate communities, reflecting the combined effects of ecological factors such as elevation, diffusion constraints, and niche differentiation. The entire area represented by the sampling sites needs conservation protection.

The niche width of common taxa is generally low. This may be related to seasonal changes in environmental factors and limitations in resource utilization capabilities.

This study provides the first systematic description of the basic composition and seasonal variation patterns of benthic macroinvertebrate communities in the high-elevation reaches of the upper Yarlung Zangbo River within Zhongba County, offering baseline data preliminary ecological inferences for future research. Subsequent studies should expand the sampling scope, increase replicate samples, and incorporate long-term environmental monitoring to more comprehensively elucidate the mechanisms driving community dynamics.

## Figures and Tables

**Table 1:** Seasonal *α*-diversity variables of the benthic macroinvertebrate community in the upper Yarlung Zangbo River.

Sampling Site	April			September		
	*H*′	*D*	*J*	*H*′	*D*	*J*
S1	0.00	0.00	0.00	2.95	2.91	0.82
S2	2.00	1.64	0.77	1.69	1.30	0.84
S3	0.99	0.51	0.99	1.37	1.24	0.86
S4	0.97	0.62	0.97	1.00	1.44	1.00
S5	1.82	1.25	0.91	1.58	1.82	1.00
S6	0.00	0.00	0.00	2.24	2.06	0.96
S7	0.99	0.51	0.99	2.42	2.01	0.94
S8	1.06	0.96	0.67	2.55	2.08	0.91
S9	0.00	0.00	0.00	0.54	0.48	0.54
S10	1.15	1.03	0.72	1.00	0.72	1.00

*H*′ = Shannon–Wiener diversity variable; *D* = Margalef richness variable; *J* = Pielou evenness variable; n = 10 per season.

**Table 2:** Seasonal niche width of common benthic macroinvertebrates in the upper Yarlung Zangbo River.

Common Taxa	Niche Width	
	April	September
*Monodiamesa* sp.	0.36	0.26
*Tanytarsus* sp.		0.64
*Orthocladius* sp.1		0.53
*Chironomus anthracinus*	0.71	
*Ilisia* sp.		0.52
*Tadamus* sp.1	0.43	
*Isoperla* sp.		0.62
*Baetis* sp.		0.41
*Apatania* sp.	0.41	
Corixidae spp.	0.32	0.68
*Nebrioporus* sp.		0.65
*Valvata* sp.	0.65	
*Piscicola geometra*		0.71
*Nais* sp.		0.48
*Gammarus* sp.		1.87

**Table 3:** Niche overlap index of common taxa in the upper Yarlung Zangbo River in April.

	Niche Overlap Index of Common Taxa in April					
Common Taxa	*Monodiamesa* sp.	*Chironomus* *anthracinus*	*Tadamus* sp.1	*Apatania* sp.	Corixidae spp.	*Valvata* sp.
*Monodimesa* sp.	1					
*Chironomus anthracinus*	0	1				
*Tadamus* sp.1	0	0	1			
*Apatania* sp.	0	0	0.33	1		
Corixidae spp.	0.12	0	0	0	1	
*Valvata* sp.	0	0.98	0	0	0	1

**Table 4:** Niche overlap index of common taxa in the upper Yarlung Zangbo River in September.

Common Taxa	Niche Overlap Index										
	*Piscicola geometra*	*Nais* sp.	*Gammarus* sp.	Corixidae spp.	*Isoperla* sp.	*Baetis* sp.	*Monodiamesa* sp.	*Tanytarsus* sp.	*Orthocladius* sp.1	*Ilisia* sp.	*Nebrioporus* sp.
*Piscicola geometra*	1										
*Nais* sp.	0	1									
*Gammarus* sp.	0.21	0.53	1								
Corixidae spp.	0.32	0	0	1							
*Isoperla* sp.	0	0	0.44	0.51	1						
*Baetis* sp.	0	0	0.38	0	0.18	1					
*Monodiamesa* sp.	0	0.62	0.27	0	0	0	1				
*Tanytarsus* sp.	0	0.67	0.33	0	0	0	0.84	1			
*Orthocladius* sp.1	0	0	0.67	0	0.77	0.28	0	0	1		
*Ilisia* sp.	0	0.50	0.85	0	0.41	0.22	0	0	0.63	1	
*Nebrioporus* sp.	0	0	0.34	0.80	0.77	0.14	0	0	0.80	0.32	1

## Data Availability

The information provided in this research can be obtained from the corresponding author upon request.
